# An adaptive map-matching algorithm based on hierarchical fuzzy system from vehicular GPS data

**DOI:** 10.1371/journal.pone.0188796

**Published:** 2017-12-05

**Authors:** Jinjun Tang, Shen Zhang, Yajie Zou, Fang Liu

**Affiliations:** 1 School of Traffic and Transportation Engineering, Key Laboratory of Smart Transport in Hunan Province, Central South University, Changsha, China; 2 School of Transportation Science and Engineering, Harbin Institute of Technology, Harbin, China; 3 Key Laboratory of Road and Traffic Engineering of Ministry of Education, Tongji University, Shanghai, China; 4 School of Energy and Transportation Engineering, Inner Mongolia Agricultural University, Hohhot, China; Beihang University, CHINA

## Abstract

An improved hierarchical fuzzy inference method based on C-measure map-matching algorithm is proposed in this paper, in which the C-measure represents the certainty or probability of the vehicle traveling on the actual road. A strategy is firstly introduced to use historical positioning information to employ curve-curve matching between vehicle trajectories and shapes of candidate roads. It improves matching performance by overcoming the disadvantage of traditional map-matching algorithm only considering current information. An average historical distance is used to measure similarity between vehicle trajectories and road shape. The input of system includes three variables: distance between position point and candidate roads, angle between driving heading and road direction, and average distance. As the number of fuzzy rules will increase exponentially when adding average distance as a variable, a hierarchical fuzzy inference system is then applied to reduce fuzzy rules and improve the calculation efficiency. Additionally, a learning process is updated to support the algorithm. Finally, a case study contains four different routes in Beijing city is used to validate the effectiveness and superiority of the proposed method.

## 1 Introduction

Large-scaled probe vehicles are recently regarded as an effective way to collect traffic data for estimating traffic conditions in urban road networks. Compared with some stationary data collection methods, such as loop detectors and video detectors, this approach provides temporal-spatial traffic information and covers a wider detecting area. The data source collected in probe vehicle system is mainly derived from Global Positioning System (GPS) equipment, which includes vehicle position, speed, and direction. Furthermore, the accuracy of data collection directly influences the reliability of analysis results. Due to the impact of surrounding buildings, tunnel or weather, positioning errors and single inference in the urban city will cause the vehicle location information from GPS devices sometimes be inaccurate. In order to overcome such positioning problem, Map-Matching (MM) algorithm is widely used to determine vehicle position on the road. This algorithm is based on combination of positioning data and digital road map, and it is considered as an effective technique to enhance positioning accuracy. Generally, map-matching algorithm includes two calculation steps: (1) to recognize the actual link vehicle driving on from all the candidate links; (2) to identify the possible location of vehicle for the matched link in the first step.

Previously, researchers have proposed a lot of effective and practical map-matching methods to solve the above problems in navigation system. Quddus et al. [1] categorized map-matching approaches into four categories: geometric analysis, topological analysis, probabilistic algorithm and advanced methods. In the geometric method, a raw GPS point is matched to the closest node or shape point, which is called point-to-point matching [2]. Another is point-to-curve matching, in which the position is matched onto the closest curve [3]. The last one is curve-to-curve matching, in which vehicle’s trajectory is simultaneously matched onto the closest link [4]. Topological map-matching methods consider geometry of the links as well as the connectivity, such as road turn, road curvature, and road connection [5–7]. The probabilistic algorithms [8–9] define error region based on an elliptical or rectangular confidence region around a position fix obtained from a navigation sensor. Advanced methods [10–13] implement some intelligent techniques such as Kalman filter, Bayesian inference, Dempster-Shafer mathematical theory and fuzzy logic, to finish map-matching process.

The result of map-matching algorithm usually suggests that vehicle is more likely to drive on one specific link than other links. Fuzzy logic, a technique for transferring qualitative terms into quantitative values, is an effective way to deal with qualitative terms such as likeliness in MM algorithms. In fuzzy logic, membership function is used to mathematically express linguistic terms with vague concepts based on fuzzy sets. A fuzzy inferences system is then constructed by set of rules to represent expert knowledge and experience [12]. Currently, a series of works are proposed to apply in the process of MM [12, 14–17]. Aim to improve calculation speed, Kim and Kim [14] proposed a C-measure map-matching algorithm based on adaptive fuzzy network (AFN). In this method, the C-measure is defined to represent the certainty of the car’s existence on the corresponding road. The convergent learning rules are then designed in the AFN to achieve high robust and matching accuracy. Quddus et al. [12] proposed an improved fuzzy logic map-matching algorithm, in which the input variables include the speed of the vehicle, the connectivity among road links, the quality of position solution, and the position of a fix relative to a candidate link. Three types of fuzzy rules are used in the inference process, including initial map-matching process, subsequent map-matching on a link, and subsequent map-matching at a junction. Zhao [15] introduced a fuzzy controller with eight fuzzy rules in the vehicular navigation with DR sensor data. Syed and Cannon [16] also developed a fuzzy logic based map-matching algorithm using GPS data. Fu et al. [17] designed a hybrid map matching method integrate fuzzy inference model and geometric features of the road network.

In this study, an adaptive map-matching algorithm based on hierarchical fuzzy system is proposed, and the contribution or improvement of the work includes following three parts: (1) Historical trajectory. Although various map-matching algorithms are introduced in the previous works, most algorithms focus on current positioning information but ignore historical trajectories. The historical trajectories of vehicle imply important information about driving routes choice. In the map-matching process of most methods only considering current positioning information, the matching results frequently tend to be inaccurate for insufficient information in urban road network with high density, especially at junctions. In order to capture characteristics of historical trajectories, this study uses a curve-curve matching method in MM algorithm, in which we define an average historical distance to evaluate the similarity between vehicle trajectory and road shape. (2) Adaptive learning scheme. We adopt the learning process in work of Kim and Kim [14]. Though designing learning rules, the model parameters can be iteratively optimized and matching accuracy will be improved. (3) Hierarchical fuzzy inference structure. In the fuzzy inference, input variables include the distance between position point and candidate links, angle between vehicle heading and candidate links, and average distance between historical trajectory and candidate links. The output variable of fuzzy inference represents the possibility to match positioning data to the candidate road. As using three input variables in the fuzzy inference system, the number of reasoning rules increases exponentially. A hierarchical fuzzy inference technique is used to simplify fuzzy rules and subsequently improve calculation efficiency of MM algorithm.

## 2 Certainty measure based map-matching algorithm

### 2.1 Definition of certainty

Kim and Kim [14] proposed an algorithm to evaluate the matching certainty of position point to candidate roads, which is defined as C-measure. In this method, two important factors, distance from projected road to vehicle position and vehicle heading angle, are used to calculate the values of certainty. This work improve the original study by adopting three factors to construct C-measure: distance between position point and candidate links, average distance between historical trajectory and candidate links, angle between vehicle heading and candidate links, which can be defined as following:

Let *p*_*c*_ = (*x*_*c*_, *y*_*c*_) presents the vehicle position and *p*_*l*_ = (*x*_*l*_, *y*_*l*_) presents the projected location on the link, ${\vec{v}}_{v}$ is the velocity, and ${\vec{v}}_{l}$ represents the projected velocity of the vehicle in the link direction. For the distance,

$$D=\frac{1}{1+\frac{{\left\|p_{c}-p_{l}\right\|}^{2}}{\delta^{2}}}$$ (1)

where *δ* means the standard deviation of the navigation filter error, *D* represents the certainty of distance between position point and candidate links, the values of *D* are in the range of [0, 1].

Similarly, for the average distance,

$$D_{ave}=\frac{1}{1+\frac{d_{ave}{}^{2}}{\delta^{2}}}$$ (2)

*d*_*ave*_ denotes average historical distance, and it means historical information (here, we consider the average distance of ten positioning points), *D*_*ave*_ represents the certainty of distance *d*_*ave*_, which also ranges between [0, 1].

For the angles,

$$A=\frac{\left({\vec{v}}_{v}\cdot {\vec{v}}_{l}\right)}{{\left\|{\vec{v}}_{v}\right\|}^{2}{\left\|{\vec{v}}_{l}\right\|}^{2}}={\text{cos}}^{2}\theta$$ (3)

where *θ* represents angle between ${\vec{v}}_{v}$ and ${\vec{v}}_{l}$, ${\vec{v}}_{v}\cdot \vec{v}$ is inner product of two vectors, *A* indicates the certainty of angle between vehicle heading and candidate links, its value is in the range of [0, 1]. Furthermore, we use *D*(*k*), *D*_*ave*_(*k*) and *A*(*k*) to represent the certainty values at the *k*th step. Thus, the improved *C*-measure map matching algorithm is updated as:

$$C(k)=\alpha_{1}D(k)+\alpha_{2}A(k)+\alpha_{3}D_{ave}(k)$$ (4)

where *α*_1_, *α*_2_, *α*_3_ are the weighting parameters, and *α*_1_>0, *α*_2_>0, *α*_3_>0. In this method, we also consider another important factor in map matching process: connectivity. As we known, when a vehicle runs from one road to another, it should go through an intersection. That is the *C*-measure at previous time period will definitely influence its value at later period. By fusing the connectivity, the algorithm is then improved as:

$$C(k+1)=\alpha_{1}D(k)+\alpha_{2}A(k)+\alpha_{3}D_{ave}(k)+\alpha_{4}C(k)$$ (5)

where *α*_4_ represents the weight of C-measure at previous moment, also *α*_4_>0.

### 2.2 Selection of threshold

In the study of [14], the map matching process includes following two modes: (1) the position-fixing mode, its aim is to determine the correct road. In this mode, if *C*(*k*) of the *i*th road is the maximum value and *C*_i_(*k*)≥*C*_T_, then it indicates that the *i*th road is the true road, *C*_T_ is the threshold in the algorithm. After identifying the correct road, the algorithm then turns to the tracking mode. (2) the tracking mode, in this mode, if *C*(*k*) of the tracked road is smaller than *C*_T_, it means that the vehicle does not run on the tracked road any more, and the *C*(*k*)s of all the roads are initialized to 0 and the algorithm will switch to the first mode. Furthermore, Kim and Kim [14] also provides two criteria to select parameters: (1) *C*(*k*) should maintain a certain value and should be less affected by the noise of the navigation filter; (2) *C*(*k*) of the correct road should be distinguished distinctly from those of other roads. The correct road is defined as the road where the vehicle actually runs on. For the first condition, we can set the value of *α*_4_ ranges in [0, 1], so the *C*-measure in Eq (5) is able to keep as a finite steady-state value. For the second condition, we can select a proper threshold *C*_T_ to distinguish *C*(*k*) of the correct road from the others. More detailed information about this algorithm can be referred to Kim and Kim [14].

**Fig 1:**
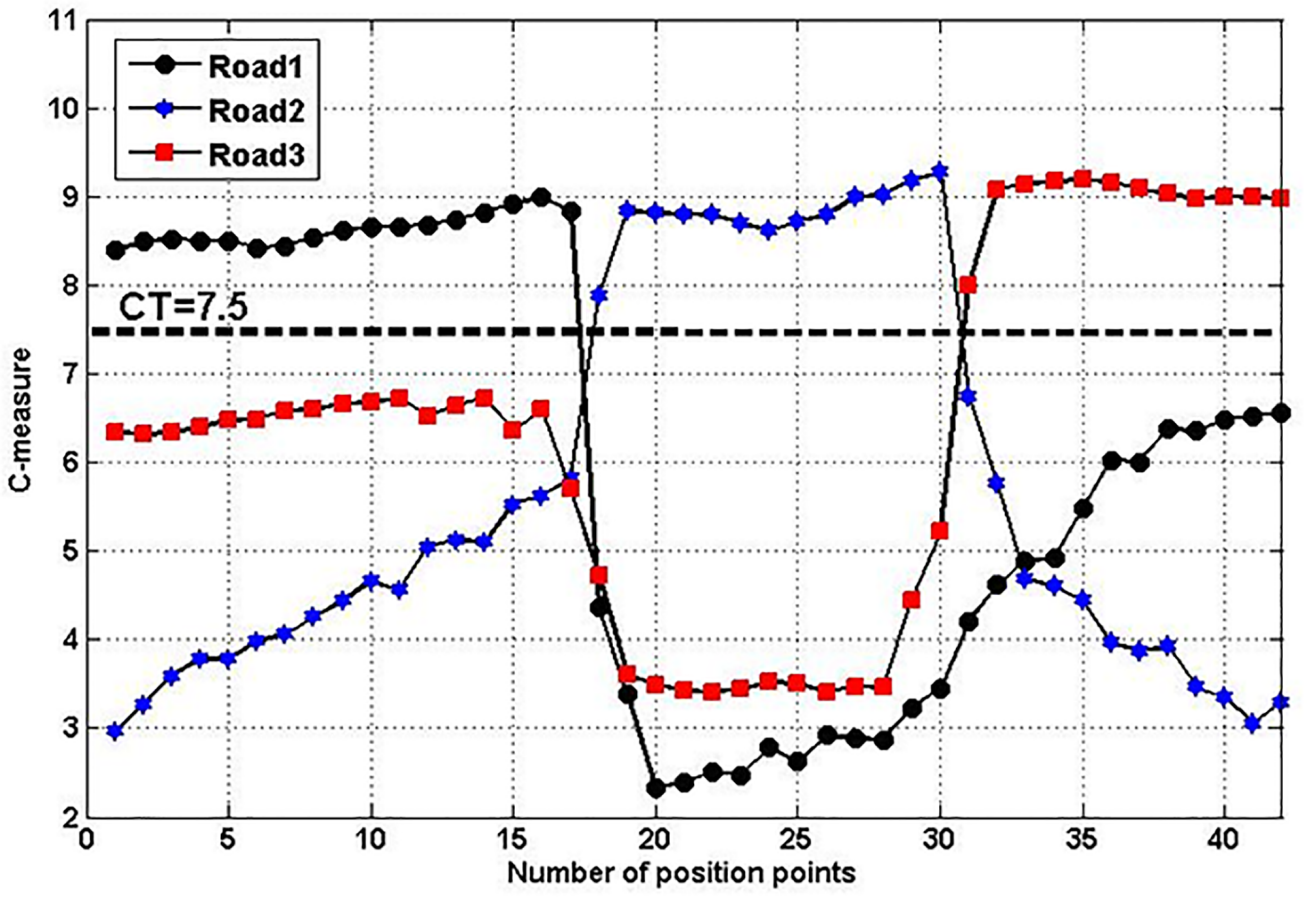
Selection of threshold *C*_T_ among three roads.

Fig 1 shows an example about how to determine *C*_T_, and we set *α*_1_ = 2, *α*_2_ = 1, *α*_3_ = 2, *α*_4_ = 0.5 from the aforementioned discussion. The figure displays C-measure variations of alternative roads. The correct road can be identified by comparing C-measure of each road between the thresholds *C*_T_. If there is a road with higher C-measure than *C*_T_, then it is will be determined as correct road on which the vehicle runs. So, if we set *C*_T_ = 7.5, *C*(*k*) of the correct road is higher than *C*_T_. The algorithm can produce correct matching results. If we increase the value of *C*_T_ to 8.5, some positions may be mismatched as *C*(*k*) of the correct road becomes lower than *C*_T_. If we select a lower value, for example, *C*_T_ = 5.0, the chance to match the wrong road becomes higher. Therefore, in order to decrease the probability for selecting wrong road, *C*_T_ is properly decided as 7.5 in the provided case.

## 3 Hierarchical fuzzy inference structures

During the learning process according to Kim and Kim [14], the *C*-measure algorithm can be rewritten as:

$$C(k+1)-\alpha_{4}C(k)=\mu (k)$$ (6)

$$\mu (k)=\alpha_{1}D(k)+\alpha_{2}A(k)+\alpha_{3}D_{ave}(k)$$ (7)

In this study, *D*, *A* and *D*_*ave*_ are the three input variables of fuzzy inference system, and output variable *y*(*k*) is used to approximately fit *u*(*k*) in Eq (6). Thus, our AFN-based method is shown as:

$$C(k+1)=\alpha_{4}C(k)+y(k)$$ (8)

where *y*(*k*) is the output of the AFN.

**Fig 2:**
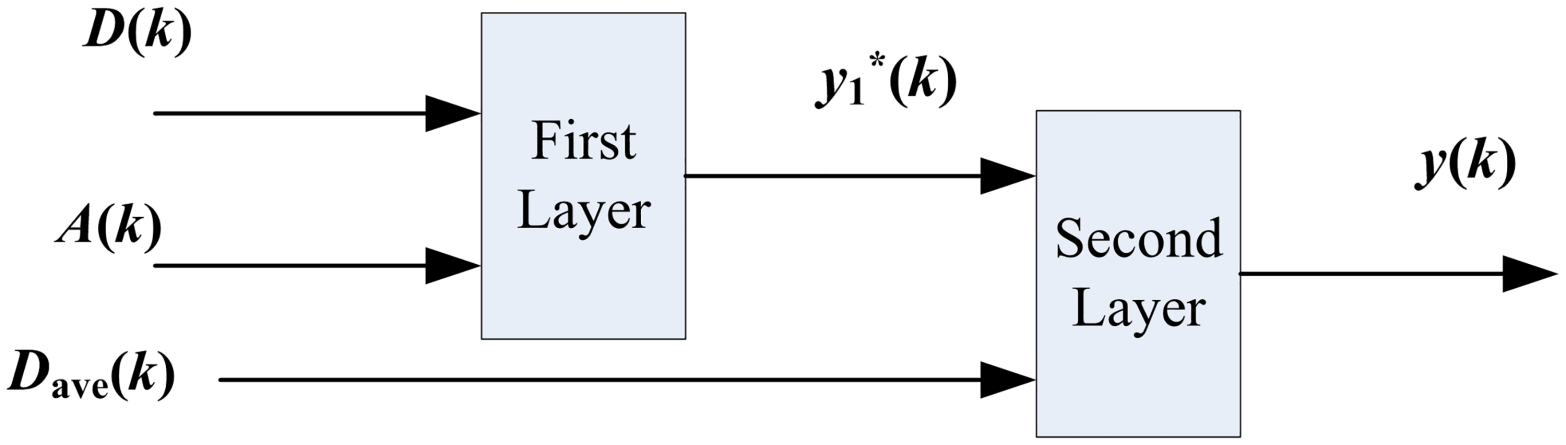
Fuzzy inference system with two layers based on historical information.

In fuzzy inference systems, the increase of input variables number will cause the number of fuzzy rules exponentially increase. Large amount of fuzzy rules would affect the efficiency of fuzzy inference system in applications. The hierarchical structure is designed to solve this problem, which was proposed by Raju et al. [18]. It comprises a number of hierarchically connected low-dimensional fuzzy systems. The input variables *D*, *A*, and *D*_ave_ all contain seven fuzzy subsets: very high (VH), higher (HER), high (HI), medium (ME), low (LO), lower (LER), and very low (VL). Fig 2 shows the hierarchical AFN structure to reduce fuzzy rules. In the first layer, *D* and *A* are input variables, and y*(*k*) is the output variable. Accordingly, y*(*k*) and *D*_ave_ are the input variables for the second layer, fuzzy subsets of y*(*k*) also have seven linguistic terms, and *y*(*k*) is the output variable. Finally, the number of fuzzy rules decreases from 7^3^ = 343 in conventional fuzzy systems to 7^2^×2 = 98 in the hierarchical structure.

**Fig 3:**
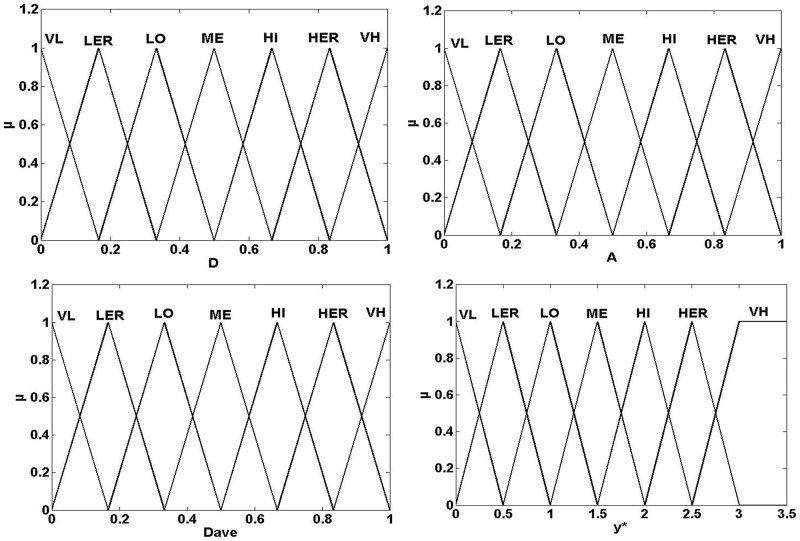
Membership functions of input variables for the hierarchical fuzzy system. (a) membership function of *D* in the first layer. (b) membership function of *A* in the first layer. (c) membership function of *D*_*ave*_ in the second layer. (d) membership function of y* in the second layer.

The defuzzified output of the network is defined as:

$$\begin{array}{l} y*(D,A)=\sum_{i=1}^{n}\mu_{D^{i}}(D)\mu_{A^{i}}(A)y_{i}^{*C}\\ =\zeta_{11}\theta_{11}+\zeta_{12}\theta_{12}+\cdots +\zeta_{1n}\theta_{1n}\\ =\zeta_{1}^{T}\theta_{1} \end{array}$$ (9)

$$\begin{array}{l} y(D,A,D_{ave})=\sum_{i=1}^{m}\mu_{y^{*i}}(y^{*})\mu_{D^{i}{}_{ave}}(D_{ave})y_{i}^{C}\\ =\zeta_{21}\theta_{21}+\zeta_{22}\theta_{22}+\cdots +\zeta_{2m}\theta_{2m}\\ =\zeta_{2}^{T}\theta_{2} \end{array}$$ (10)

where *θ*_1_^T^ = [*y*_1_*^C^ *y*_2_*^C^…*y*_n_*^C^] represents the parameters vector to be optimized, *n* indicates the number of rules in the first layer, *y*_i_*^C^ represents the center of membership function in reference rules of first layer, *ζ*^T^_1_(*D*, *A*) = [*ζ*_11_ *ζ*_12_*…ζ*_1n_] is a vector of static terms and *ζ*_1i_ represents the product of membership to *D* and *A*. Similarly, *θ*_2_^T^ = [*y*_1_^C^ *y*_2_^C^*…y*_n_^C^] also represents the parameters vector should to be optimized, *m* denotes the number of rules in the second layer, *y*_i_^C^ represents the center of membership function in reference rules of second layer, *ζ*^T^_2_(*D*_*ave*_, y*) = [*ζ*_21_ *ζ*_22_*…ζ*_2m_] is a vector of static terms and *ζ*_2*i*_ represents the product of membership to y* and *D*_*ave*_. Fig 3 shows the membership function of input variables in two fuzzy inference layers. The reasoning rules in the first and second layer are provided in Tables 1 and 2 respectively, and the numbers of rules are both 49.

**Table 1:** Fuzzy reasoning rules in the first layer.

*A*	VL	LER	LO	ME	HI	HER	VH
*D*							
VL	VL	VL	LER	LER	LER	ME	ME
LER	VL	LER	LER	LO	ME	ME	HI
LO	LER	LER	LO	LO	ME	HI	HI
ME	LER	LO	LO	ME	HI	HI	HER
HI	LER	ME	ME	HI	HI	HER	HER
HER	ME	ME	HI	HI	HER	HER	VH
VH	ME	HI	HI	HER	HER	VH	VH

**Table 2:** Fuzzy reasoning rules in the second layer.

*D*_ave_	VL	LER	LO	ME	HI	HER	VH
y*							
VL	VL	VL	LER	LER	LER	ME	ME
LER	VL	VL	LER	LO	ME	ME	HI
LO	LER	LER	LO	LO	ME	HI	HI
ME	LER	LO	LO	ME	HI	HI	HER
HI	LER	ME	ME	HI	HI	HER	HER
HER	ME	ME	HI	HI	HER	VH	VH
VH	ME	HI	HI	HER	HER	VH	VH

Define {*D*(*k*), *A*(*k*), *D*_*ave*_(*k*), *Y*(*k*)} as a training set, in which *D*(*k*), *A*(*k*) and *D*_*ave*_(*k*) are input variables of fuzzy inference system, and *Y*(*k*) is considered as desired output of system. The learning process can be shown follows:

$$\Delta \theta_{1}(k-1)=\eta_{1}\cdot \varepsilon (k-1)\cdot \zeta_{1}(D,A)$$ (11)

$$\Delta \theta_{2}(k-1)=\eta_{2}\cdot \varepsilon (k-1)\cdot \zeta_{2}(D_{ave},y^{*})$$ (12)

$$\theta_{1}(k)=\theta_{1}(k-1)+\Delta \theta_{1}(k-1)$$ (13)

$$\theta_{2}(k)=\theta_{2}(k-1)+\Delta \theta_{2}(k-1)$$ (14)

where *η*_1_>0, *η*_2_>0 and *ε*(*k*) = *Y*(*k*)- *y*(*k*). However, according to the study of *C*-measure method in Kim and Kim [14], in the map matching process, there is no reference model can be used to implement the above learning rules. As the purpose of learning algorithm is to find proper *θ*_1_(*k*) and *θ*_2_(*k*) to satisfy following two conditions: (1) *C*(*k*) of the correct road should be higher than other candidate roads so that the correct road can be identified; (2) *C*(*k*) of the correct road should be higher than the threshold *C*_T_. Thus, the learning rules are modified as follows:

$$\Delta \theta_{1}(k)=\{\begin{array}{cc} 0 & ifC_{m}(k)\geq C_{T}\\ \eta_{1}(\zeta_{1m}-\zeta_{1i}) & ifC_{m}(k)<C_{i}(k)\\ \eta_{1}\zeta_{1m} & ifC_{m}(k)<C_{T} \end{array}$$ (15)

$$\Delta \theta_{2}(k)=\{\begin{array}{cc} 0 & ifC_{m}(k)\geq C_{T}\\ \eta_{2}(\zeta_{2m}-\zeta_{2i}) & ifC_{m}(k)<C_{i}(k)\\ \eta_{2}\zeta_{2m} & ifC_{m}(k)<C_{T} \end{array}$$ (16)

$$\theta_{1}(k)=\theta_{1}(k-1)+\Delta \theta_{1}(k-1)$$ (17)

$$\theta_{2}(k)=\theta_{2}(k-1)+\Delta \theta_{2}(k-1)$$ (18)

where *η*_1_>0, *η*_2_>0. The *m*th road is the correct road. The initial values of *θ*_1_ and *θ*_2_ can be set as constant.

## 4 Applications and discussion

The GPS data we used in this study were collected from vehicular navigation equipment in Beijing city during seven days from Feb. 2nd to 8th in 2008. Any personal information about drivers is completely deleted, and the data were analyzed anonymously. Each car is equipped by GPS device and data are collected when the car is running on the road. The GPS data samples can be referred to the website: T-Drive trajectory data sample [19]. We used GPS data from 100 selected drivers, which are uploaded as S1 Data. In the model application, we divide data set into two parts: training dataset and testing dataset, training dataset contain one half data samples collected from 100 drivers in one week to optimize parameters in model, and testing dataset from another half data samples are used to validate the effectiveness of the proposed map-matching algorithm. Each data sample contains information collecting time and location: “Time”, “Latitude”, “Longitude”. The “Time” indicates when the data were recorded, and “Latitude” and “Longitude” provide the location information of vehicle.

In the application, we only choose four different routes in the testing dataset to validate map-matching algorithm, they have different number of GPS points and route length. Before employing validation, we should select proper parameters for each route, which are shown in Table 3 using the similar approach introduced in sub-section 2.2. Furthermore, the number of position points and length of four routes are also provided in Table 3.

**Table 3:** Basic information of testing routes.

Routes	Number	Length (km)	Parameters				
			*α*_1_	*α*_2_	*α*_3_	*α*_4_	*C*_T_
**A**	274	20.36	2	1	2	0.5	8.5
**B**	168	13.80	2	1	2	0.5	8.5
**C**	317	28.88	1.5	1	1.5	0.5	7.0
**D**	282	25.11	1.5	1	1.5	0.5	7.5

For routes A and B in the area with high density of network, the C-measures of candidate roads sometimes will become close, for example, the roads have similar patterns or parallel roads with small distance. Thus, if we use low value of *C*_T_, the C-measure of the correct road cannot be identified obviously from the other candidate roads, which will result in mismatch of position points. For routes C and D in the area with low density of network, even if a low *C*_T_ can be effectively used to distinguish true road and other candidate roads.

Table 4 shows the performance comparison between the algorithm proposed in this study with some traditional methods: the point-to-point matching algorithm [2] in geometric method, a weighted topological analysis [6] in topological method and original Adaptive Fuzzy Network (AFN) based C-measure map-matching algorithm [14] in advanced method. An indicator, ratio of correctly identified links, is defined to evaluate the accuracy of the method as follows:

$$r(\%)=\frac{l(ture)}{l(trajectory)}\times 100$$ (19)

where *trajectory* is the set of links in the actual routes, *true* is the set of links that can be correctly identified, and *l*(*x*) is the total length of the links in set *x*, *r* is the percentage of correctly identified links.

**Table 4:** Comparisons of results between different map-matching algorithms.

Map-matching algorithms	Ratio of correctly identified links (*r*:%) of four routes			
	A	B	C	D
**Geometric method**	73.57	75.34	80.52	81.76
**Topological method**	84.73	85.69	88.27	89.33
**Original method based on AFN**	92.53	91.95	95.23	94.65
**Improved method based on AHFN**	94.38	95.47	97.02	96.81

AHFN: Adaptive Hierarchical Fuzzy Network

From the validation results, we can obtain the following findings:

1. For all the algorithms, the matching performance in area with low network density is evidently higher than that of high dense network. In routes A and B, the density of roads network is high, the shapes of roads in this area express similar pattern, and distance among these roads are small. In routes C and D, as the number of candidate roads decreases, it becomes easier to recognize the true roads.
2. The improved method is superior to the original method. In the original method, after vehicle goes through the intersection, the algorithm turns to the position fixing mode. As the roads in some areas of an urban city have similar shape, and they are also parallel and close, it is difficult for original algorithm to recognize which candidate road is the correct one in the position fixing mode. Thus, under the circumstances, it is possible to determine an incorrect road. After implementing position fixing mode, the algorithm will transfer to tracking mode and start learning procedure. That is, before reaching the next intersection, the remaining vehicle position points will be matched to the same roads. Unfortunately, this mismatching result cannot be detected. In this study, we add historical certainty in algorithm to complete matching between vehicle trajectories and road shape. It effectively enhances the stability and accuracy of map-matching algorithm.
3. The algorithms using the proposed advanced methodology outperform the traditional algorithms using only geometric or topological information of network. Compared to two traditional algorithms, the AFN based map-matching algorithm not only considers connectivity of road network but also fuses the distance between position points to candidate roads, and angles between driving direction and road shape. Furthermore, the learning process can also improve matching accuracy.

## 5 Conclusions

Fusing multisource data collected from fixed detectors [20–22] and mobile sensors [23–27] to evaluate traffic states has become a key step to accomplish the smart management and control in urban city. How to match mobile or probe vehicles onto the road network is a basic work in data fusion. In this study, we proposed an improved map-matching algorithm. Firstly, we used the average historical distance to complete curve-curve matching process between vehicle trajectories and road shape. In the fuzzy inference system, the distance between position point and candidate roads, angle between driving heading and road direction, and average historical distance were regarded as input variables. As the fuzzy rules would increase exponentially with the number of variables, we then adopted the hierarchical fuzzy inference system to simplify fuzzy reasoning, in which a two layer framework was constructed. Finally, a learning scheme was designed to optimize and update parameters and threshold in proposed algorithm. In the case study, we selected four routes in Beijing city as examples to validate model. Two routes were in the area with high dense network and other two routes were in the area with low dense network. We compared the matching performance of the proposed algorithm with some traditional methods: geometric method, a weighted topological method and original C-measure map-matching algorithm. We found that the proposed algorithm in this study shows highest matching accuracy and the advanced method outperformed geometric or topological method. Furthermore, better matching performance can be obtained for routes in network with low density compare to routes in high dense network.

## Supporting information

S1 Data GPS data from 100 selected drivers. (ZIP)
